# Rapid decline in pH of coral calcification fluid due to incorporation of anthropogenic CO_2_

**DOI:** 10.1038/s41598-017-07680-0

**Published:** 2017-08-09

**Authors:** Kaoru Kubota, Yusuke Yokoyama, Tsuyoshi Ishikawa, Atsushi Suzuki, Masao Ishii

**Affiliations:** 1 Atmosphere and Ocean Research Institute, The University of Tokyo, Chiba, 277-8564 Japan; 2 Kochi Institute for Core Sample Research, Japan Agency for Marine-Earth Science and Technology, Nankoku, Kochi 783-8502 Japan; 3 Department of Biogeochemistry, Japan Agency for Marine-Earth Science and Technology, Yokosuka Kanagawa, 237-0061 Japan; 4 Geological Survey of Japan, National Institute of Advanced Industrial Science and Technology, Tsukuba, Ibaraki 305-8567 Japan; 5 Oceanography and Geochemistry Research Department, Meteorological Research Institute, Japan Meteorological Agency, Tsukuba, Ibaraki 305-0052 Japan

## Abstract

Marine calcifying organisms, such as stony corals, are under threat by rapid ocean acidification (OA) arising from the oceanic uptake of anthropogenic CO_2_. To better understand how organisms and ecosystems will adapt to or be damaged by the resulting environmental changes, field observations are crucial. Here, we show clear evidence, based on boron isotopic ratio (δ^11^B) measurements, that OA is affecting the pH of the calcification fluid (pH_CF_) in *Porites* corals within the western North Pacific Subtropical Gyre at two separate locations, Chichijima Island (Ogasawara Archipelago) and Kikaijima Island. Corals from each location have displayed a rapid decline in δ^11^B since 1960. A comparison with the pH of the ambient seawater (pH_SW_) near these islands, estimated from a large number of shipboard measurements of seawater CO_2_ chemistry and atmospheric CO_2_, indicates that pH_CF_ is sensitive to changes in pH_SW._ This suggests that the calcification fluid of corals will become less supersaturated with respect to aragonite by the middle of this century (pH_CF_ = ~8.3 when pH_SW_ = ~8.0 in 2050), earlier than previously expected, despite the pH_CF_-upregulating mechanism of corals.

## Introduction

The pH of the surface seawater (pH_SW_) is considered to have declined by ~0.1 since the beginning of the industrial era, and an additional decline of ~0.3 is projected by the end of this century^1–3^. It has been suggested that coral reef ecosystems are susceptible to reductions in the pH and aragonite saturation state of seawater (Ω_arSW_)^4–6^, although the critical threshold below which reef growth will be hampered is still contested^4, 7–9^. In the surface layers of tropical–subtropical oceans, Ω_arSW_ is projected to decrease to as low as 3 by 2050, when atmospheric CO_2_ will reach ~500 μatm^1, 4^.

**Figure 1:**
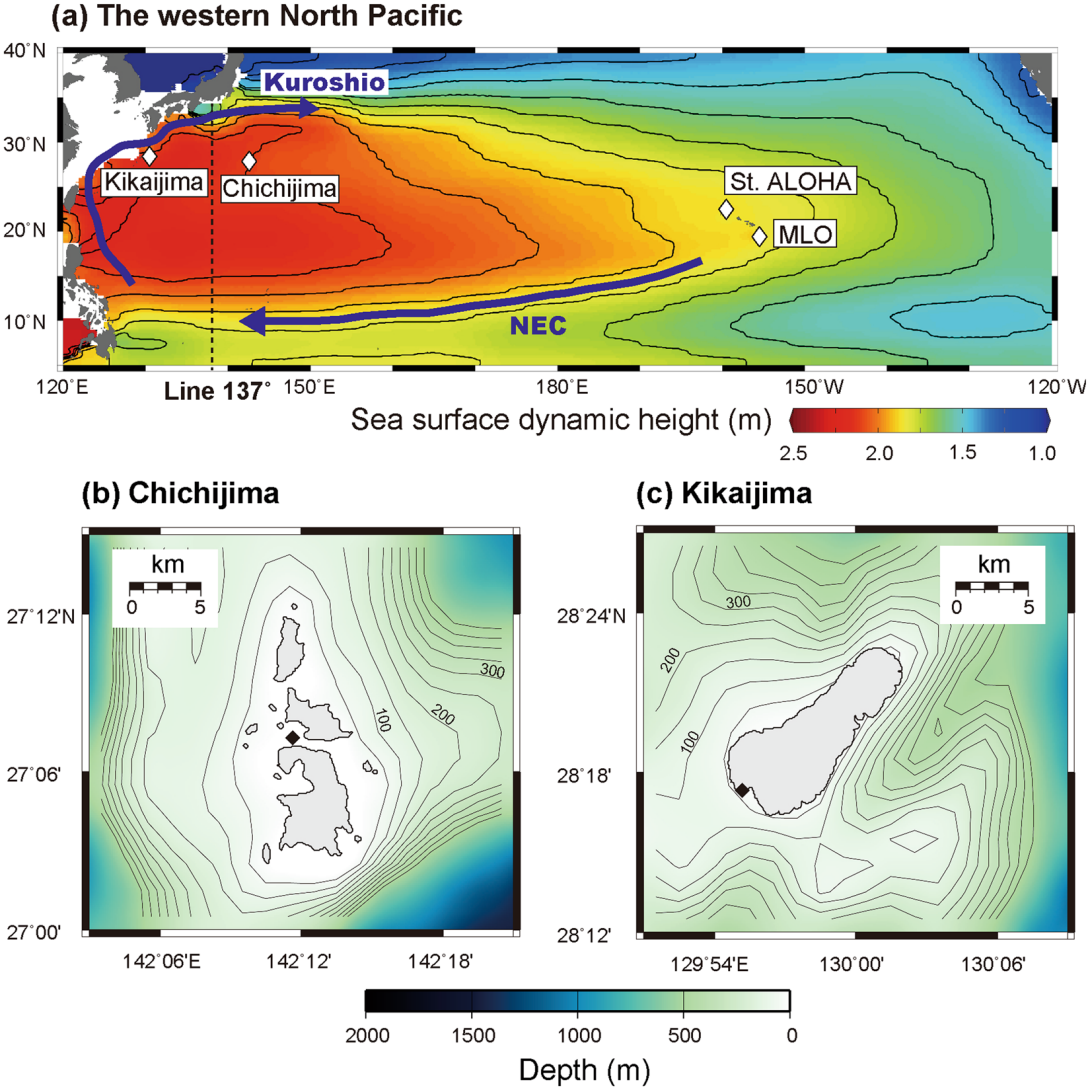
Location of Chichijima and Kikaijima in the North Pacific Ocean. (**a**) Contours indicate climatological mean sea-surface dynamic height (in units of metres relative to the 1000 m level), with arrows showing the major surface ocean currents in the western North Pacific. Data were downloaded and plotted with the ODV software, version 4.6.2 (ref. 43, http://odv.awi.de). MLO, Mauna Loa Observatory. (**b**,**c**) Bathymetric maps around Chichijima and Kikaijima. Depth contours are at 50 m intervals. The GMT software, version 4.5.8 (ref. 44), was used to map the bathymetric data, ETOPO1 (ref. 45, https://www.ngdc.noaa.gov/mgg/global/global.html). The corals were collected at a location with good open ocean seawater circulation (black dot) (Supplementary Figs S7 and S8)^10, 11^.

To better understand the response of corals to ocean acidification, we reconstructed past pH changes using skeletal δ^11^B (Methods; Fig. 1b) as an indicator of the pH of the calcification fluid (pH_CF_) in long-living massive *Porites* corals obtained from the islands of Chichijima (27.1°N, 142.2°E)^10^ and Kikaijima (28.3°N, 130.0°E)^11^. These corals have experienced OA since the beginning of the industrial revolution (ca. 1750), and particularly during the past 50 years, following the post-1960s increase in anthropogenic CO_2_ emissions. An advantage of field-based observations over culture experiments is that the latter often subject corals to excessively acidic water (e.g., pH < 7.8), which are not expected to occur this century, even under the “business as usual” CO_2_ emissions scenario (e.g., refs 12 and 13).

**Figure 2:**
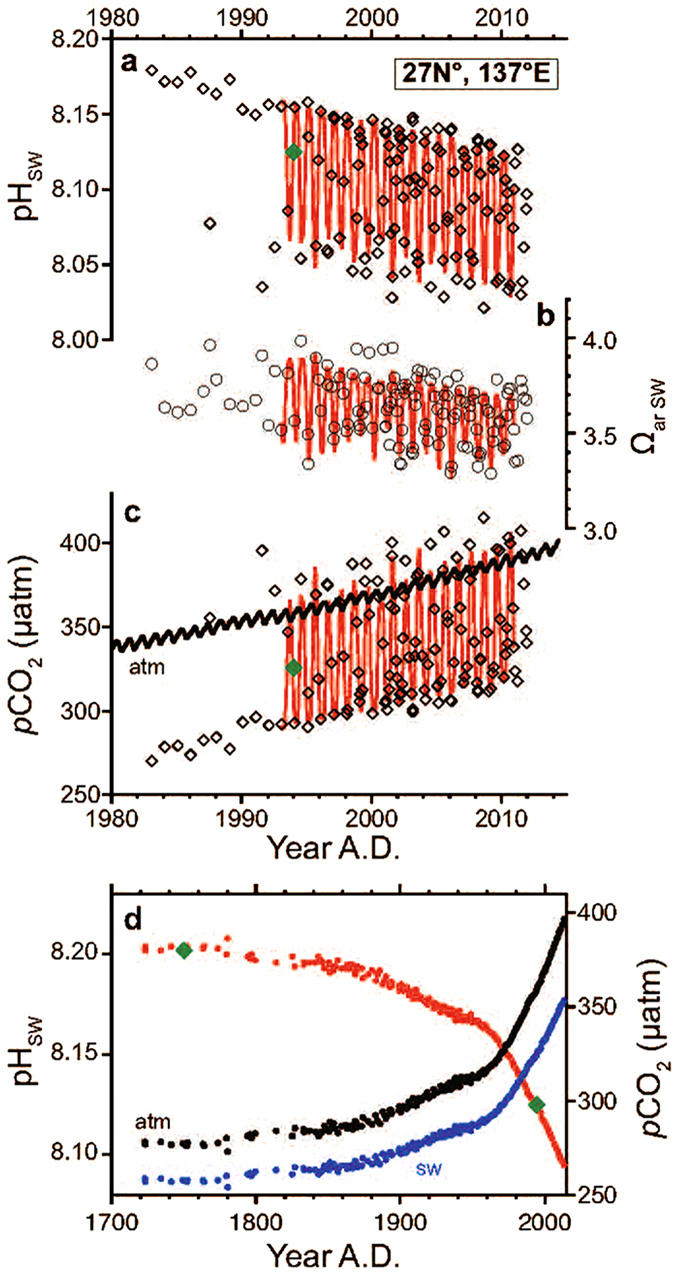
pH_SW_ and *p*CO_2_ variability. (**a**–**c**) Time series (lines) and discrete data (open symbols) for pH_SW_, Ω_arSW_, and *p*CO_2_ in the western North Pacific near Chichijima and Kikaijima since 1980 at monthly resolution. Monthly atmospheric *p*CO_2_ records measured at the MLO^17^ are also shown in (**c)**. (**d**) Variability in pH_SW_ (red) and *p*CO_2_ (blue, seawater; black, atmosphere) since the preindustrial era. Atmospheric *p*CO_2_ in 1959–2013 was measured at the MLO^17^ and atmospheric *p*CO_2_ before 1959 was reconstructed from trapped air in the Antarctic ice sheet^18^. pH_SW_ calculated from Global Data Analysis Project DIC and TA for the years 1994 and 1750 (ref. 3) are indicated by the green diamonds in (**a** and **d**).

Measurements of the seawater CO_2_ chemistry have been made in the vicinity of these two islands for the last three decades (Methods, Fig. 2). The ocean surrounding the islands is oligotrophic, with limited vertical mixing and low biological productivity^14^. Seasonality dominates the temporal variability in pH_SW_ at these locations, driving changes in the partial CO_2_ pressure of the seawater (*p*CO_2SW_) (Fig. 2a,c), primarily through variability in the sea-surface temperature (SST; ~20 °C in winter and ~29 °C in summer), and changes in the dissolved inorganic carbon (DIC) concentration (~1960 µmol kg^−1^ in summer and ~1990 µmol kg^−1^ in winter in 2010 when normalized to a salinity of 35)^15^. The increasing trend in *p*CO_2SW_ (and the decreasing trend in pH_SW_) in the northern subtropical zone of the western North Pacific follows the rate of increase in atmospheric CO_2_ (Fig. 2c)^14, 16^. Because the air–sea CO_2_ equilibrium has remained unchanged since preindustrial times, the estimated pH_SW_ can be extended back to the preindustrial era (Fig. 2d) using atmospheric CO_2_ records from the Mauna Loa Observatory (MLO, Fig. 1a)^17^ and the Antarctic ice sheet^18^.

## Results and Discussion

**Figure 3:**
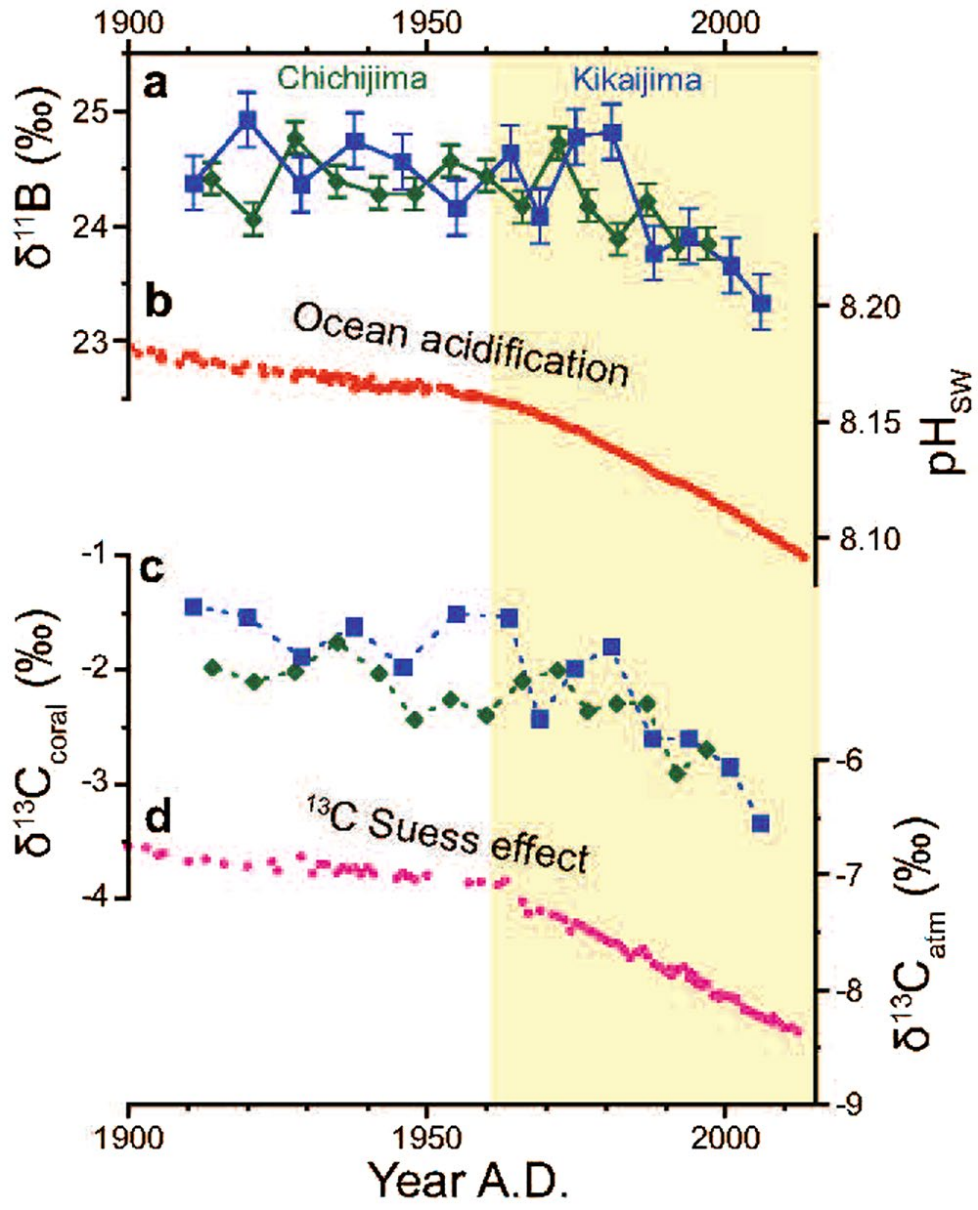
Coral δ^11^B and δ^13^C records, pH_SW_, and δ^13^C of atmospheric CO_2_. (**a**) δ^11^B records of the corals from Chichijima (green diamonds) and Kikaijima (blue squares). Each point is a 3-year average. Error bar is 2σ of the analytical uncertainty of JCp-1. (**b**) As in Fig. 2d, but for years 1900–2013. (**c**) As in (**a**) but for δ^13^C_coral_ at Chichijima^10^ and Kikaijima. δ^13^C_coral_ decreased at the same rate as δ^13^C_atm_ after 1960 (black regression line). (**d**) δ^13^C_atm_ in 1981–2012 was measured at the MLO and the values before 1981 were reconstructed from trapped air in the Antarctic ice sheet^18^.

Statistically insignificant variations in δ^11^B were recorded for the period before 1960, whereas a rapid decline in δ^11^B occurred after 1960 (−0.18 ± 0.04‰/decade for the Chichijima coral, p < 0.001; −0.29 ± 0.07‰/decade for the Kikaijima coral, p < 0.01). This trend correlates with the trend in pH_SW_ evaluated from the time series record of the atmospheric CO_2_ concentration, and the correlation is derived from a long-term decreasing trend, not inter-annual variability (Fig. 3a,b). Kubota *et al*.^19^ demonstrated that the coral skeleton δ^11^B from Chichijima Island follows the trend in ocean acidification during the 20th century, and we confirmed their initial finding by measuring δ^11^B in a second massive *Porites* coral skeleton collected from nearby Kikaijima Island. The stable carbon isotopic ratios of the corals (δ^13^C_coral_) behave in a similar way (Fig. 3c), decreasing slightly or remaining steady until 1960 (−0.11 ± 0.02‰/decade for the Chichijima coral, p < 0.001, N = 49; −0.04 ± 0.06‰/decade for the Kikaijima coral, p = 0.53, N = 6), and declining sharply thereafter (−0.25 ± 0.04‰/decade for the Chichijima coral, p < 0.01, N = 35; −0.34 ± 0.08‰/decade for the Kikaijima coral, p < 0.01, N = 8). These patterns in δ^13^C_coral_ are consistent with the previously reported records of stable carbon isotopic ratios for atmospheric CO_2_ (δ^13^C_atm_), corresponding to −0.04 ± 0.01‰/decade (p < 0.01) before 1960 and −0.24 ± 0.01‰/decade (p < 0.01) after 1960 (ref. 18) (Fig. 3c,d). Such recent reductions in the δ^13^C of the carbon reservoir in the Earth surface system is called the ‘^13^C Suess effect’^20^, and are caused by the anthropogenic addition of ^12^C-rich carbon, derived from fossil fuel burning and deforestation, to the Earth surface system. The recent reductions in δ^13^C_coral_ in the Kikaijima and Chichijima corals are consistent with the expected reductions attributable to the^13^C Suess effect, which has been observed directly in atmospheric CO_2_ (ref. 18) and the δ^13^C_DIC_ of the surface seawater in the subtropical North Pacific^21, 22^. Other potential drivers of δ^13^C_coral_ and δ^11^B are the growth rate of the skeleton^23^ and the photosynthetic activity of algal symbionts^24^. We argue these effects on δ^13^C_coral_ are minor compared with the^13^C Suess effect, for the following reasons. If the decline in δ^13^C_coral_ is solely explained by changes in the growth rate, it would require a substantial increase in the growth rate (e.g., from 5 mm/yr to 10 mm/yr). However, it is inconsistent with the observation that both the Chichijima coral and Kikaijima coral show no increase in their growth rates^10, 11^. We infer that the correlation between the annual δ^13^C variation and the linear extension rate of the Chichijima coral reported by Felis *et al*.^10^ can be interpreted as the growth rate effect superimposed on the^13^C Suess effect. During photosynthesis, symbiotic algae preferentially utilize isotopically lighter carbon and leave isotopically heavier carbon in the carbon pool from which the corals precipitate their skeletons^13, 24^. The recent declines in δ^13^C_coral_ have been interpreted as reflecting substantial reductions in light intensity or photosynthetic activity. However, a previous culture experiment with *Porites* coral^24^ indicated that this would require a reduction in light intensity of >50%, which is unlikely to occur in the shallow waters where corals live. We also observed significant positive correlations between δ^11^B (and pH_CF_) and δ^13^C_coral_ (r = 0.63, p < 0.03 for the Chichijima coral; r = 0.85, p < 0.001 for the Kikaijima coral; Supplementary Fig. S2). Therefore, we conclude that the coherent declines in δ^11^B and δ^13^C in the Chichijima and Kikaijima corals resulted from reductions in pH_SW_ and δ^13^C_DIC_, respectively. This suggests that the declines in both δ^11^B and δ^13^C_coral_ are anthropogenic in origin, and that fingerprints of anthropogenic CO_2_ uptake by the ocean (OA and the^13^C Suess effect, respectively) are recorded in the coral skeleton. The probability that the reductions in both δ^11^B and δ^13^C are caused by local factors is very small, for the following reasons: no rivers bring organic matter from the land at these sites, which would cause local acidification and lower the δ^13^C_DIC_ when it is degraded; and there is no coastal upwelling around these islands that would acidify the subsurface water and lower its δ^13^C_DIC_. We directly compared the geochemical record of the corals with the open ocean CO_2_ chemistry, because they were collected from locations that receive good open ocean seawater circulation (Methods). However, the seawater CO_2_ chemistry may be locally modified by the net community calcification/respiration of the coral reef ecosystems, and we did not confirm this by measuring the variables of the seawater CO_2_ system. Even if these are local signals, and are not related to the CO_2_ chemistry of the open ocean seawater, the community calcification/respiration in these coral reef ecosystems has still changed. If so, this intriguing observation gives many clues to the local ecosystem. However, as stated above, we infer that the reductions in both δ^11^B and δ^13^C are related to changes in the CO_2_ chemistry of the open ocean, based on geographic observations at the study sites.

**Figure 4:**
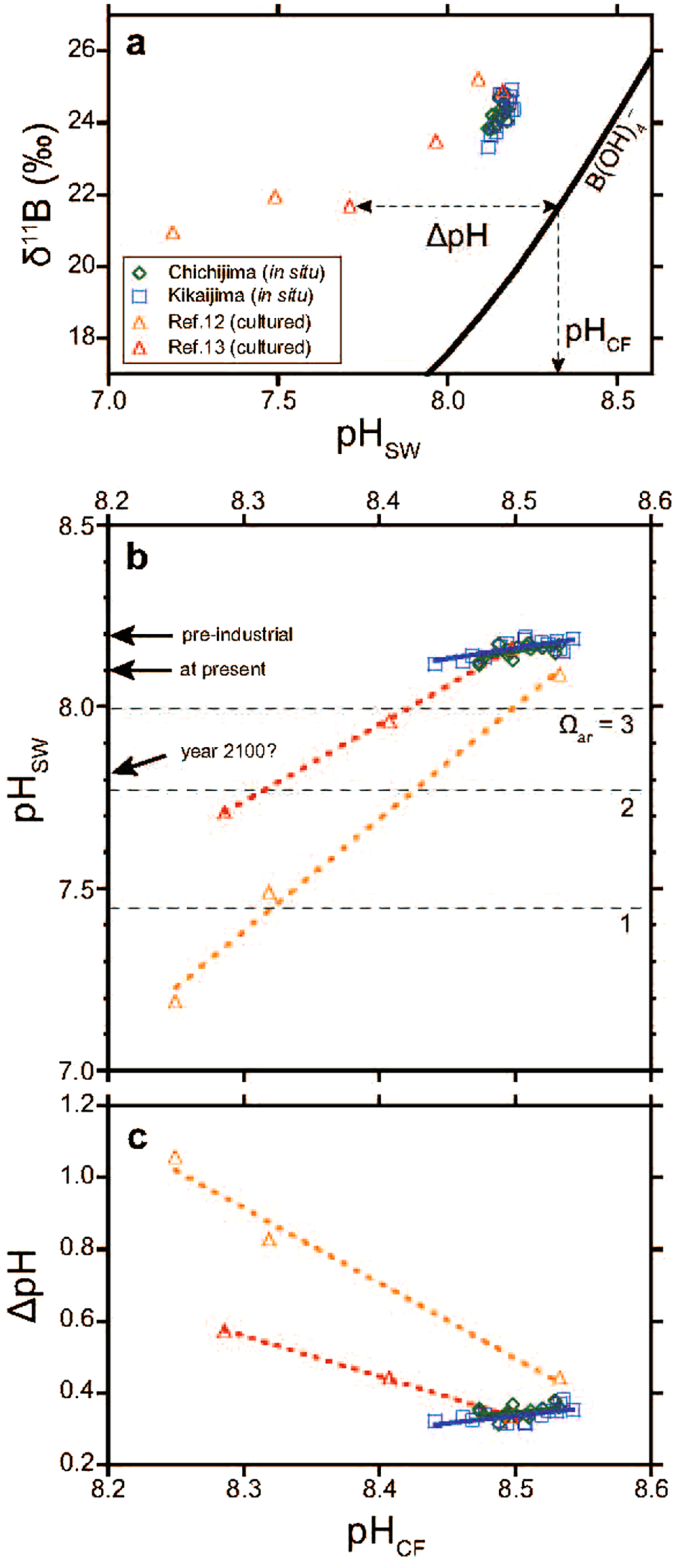
Relationships between pH_SW_ and pH_CF_. (**a**) δ^11^B values for long-living and cultured corals (green diamonds, Chichijima *Porites* sp.; blue squares, Kikaijima *Porites* sp.; yellow triangles, cultured *Porites* sp., ref. 12; red triangles, cultured *Porites cylindrica*, ref. 13) with the theoretical curve for δ^11^B of the borate ion in seawater^25^. With the pH-upregulating mechanism, δ^11^B records not pH_SW_ but pH_CF_, and ΔpH represents pH_CF_ minus pH_SW_. (**b**) A cross-plot of pH_CF_ versus pH_SW_. Regression lines are shown for each coral record. Dashed horizontal lines indicate pH values when Ω_ar_ becomes 3, 2, and 1, which were calculated using present SST, SSS, and TA. (**c**) As in (**b**) but for pH_CF_ versus ΔpH.

We observed large differences between pH_SW_ (8.12–8.18), determined from measurements of the CO_2_ chemistry, and pH_CF_ (8.43–8.53), derived from the δ^11^B of the corals and a theoretical curve for δ^11^B of the borate ion^25^ (Fig. 4a,b and Supplementary Table 1). One plausible explanation for the fact that pH_CF_ is higher than pH_SW_ is the proposed “pH upregulation mechanism” (refs 6, 26–28). The (extracellular) calcification fluid occurs between the coral polyp and the underlying skeleton, and so is semi-isolated from the ambient seawater. Corals use Ca^2+^-ATPase to pump H^+^ from and Ca^2+^ into the calcification fluid, which in turn increases pH_CF_ and Ω_ar_ (Ω_arCF_), thus promoting calcification^26–28^. *In situ* pH_CF_ measurements using micro-pH electrodes and pH-sensitive dyes support the idea of pH upregulation^6, 26, 27^. pH_CF_ has been estimated in *Stylophora pistilata* with pH-sensitive dyes and δ^11^B measurements, which were in excellent agreement assuming three times faster calcification in the light than that in the dark^29^. Because light-enhanced calcification probably creates a certain bias in the time of maximum calcification (i.e., day time in summer), the δ^11^B of the *Porites* corals in this study represents a mean state of pH_CF_, unless the time of maximum calcification changes. The periodic patterns in the seasonality of geochemical proxies, such as the Sr/Ca ratio, in the Chichijima and Kikaijima coral skeletons do not support any marked changes in the growth season^10, 11^. Therefore, as in previous studies^26, 28, 29^, we regard the δ^11^B of the *Porites* corals as representative of pH_CF_ on average, at a given pH_SW_. It has been suggested that *Porites* corals, as well as other scleractinian coral genera, can use their pH-upregulating mechanism to maintain their pH_CF_ under ongoing OA^26–28^. Therefore, corals are able to increase ΔpH to maintain pH_CF_ as high as possible, perhaps by maintaining the homeostasis of the calcification fluid (Fig. 4a).

The relationship between pH_SW_ and pH_CF_ in two cultured *Porites* corals^12, 13^ predicts reductions of only 0.03 and 0.05 in pH_CF_ per reduction of 0.1 in pH_SW_ (Fig. 4b), which suggests that coral δ^11^B may not be sensitive enough to detect anthropogenic OA. However, we detected a clear indication of a rapid decline in pH_CF_ in both the Chichijima and Kikaijima corals with the decline in pH_SW_ (p < 0.03), which was not observed in the cultured corals (Fig. 4b,c). The observation of a one-to-one relationship between pH_CF_ and pH_SW_ strengthens the reliability of the δ^11^B of *Porites* coral as a proxy for pH_SW_, although *in situ* calibration is crucial^19, 30^. Although the ΔpH values are similar (0.3–0.5) among the corals in the field and in the culture environments in the present pH_SW_ range of 8.09–8.17 (Fig. 4c), the rates of ΔpH for both cases differ significantly (p < 0.01) (Fig. 4c). We also found that the changes in pH_CF_ were more sensitive to OA in the Chichijima and Kikaijima corals than in any other previously cultured scleractinian corals reported to date (e.g., *Acropora*, *Stylophora*, and *Cladocora*; p < 0.03), and their pH_CF_ was estimated from δ^11^B (ref. 26). A recent study found that the pH_CF_ of the branching coral *Porites cylindrica* cultured on Heron Island, on the Great Barrier Reef, was unaffected by a reduction in pH_SW_^28^, which contradicts our observations on Chichijima and Kikaijima Islands. That study inferred that the highly variable conditions of the seawater CO_2_ chemistry at that location caused the pH_CF_ of the corals to be more resilient to OA. If so, the more sensitive declines in pH_CF_ seen in the Chichijima and Kikaijima corals may be attributable to seawater in which the CO_2_ chemistry is less variable than that affecting large coral reefs such as the Great Barrier Reef. This suggests that colonies in similar environments may be more susceptible to OA.

Because corals expend energy when upregulating pH_CF_, maintaining a constant ΔpH (Fig. 4c) seems reasonable from the perspective of biological adaptation^5, 6, 26^. However, lower pH_CF_ under OA leads to the slower calcification of corals^26^, increasing their susceptibility to bio-erosion by grazers and burrowers, and perhaps creating a competitive disadvantage relative to organisms such as macroalgae^31^. Because the Ca^2+^ concentration in the calcification fluid is almost the same as that in seawater (<10% change)^6, 29^, an sensitive decline in pH_CF_ will lead to a faster decline in Ω_arCF_, because pH_CF_ regulates Ω_arCF_ (Supplementary Section 1 and Supplementary Fig. S3 and Supplementary Table S2). Therefore, it is reasonable to infer that OA overwhelms or disables the pH_CF_ homeostasis of the coral, rather than that the coral spontaneously regulates pH_CF_. Although we observed no long-term changes in the linear extension rate of the Chichijima and Kikaijima coral skeletons during the last 100 years (Supplementary Fig. S1; refs 10 and 11), this may be attributable to the stretch modulation of corals because some stony corals maintain a linear skeletal extension rate in a stressed environment, at the expense of skeletal density (e.g., ref. 8).

As well as pH_SW_ and Ω_arSW_, temperature is also an important factor for corals because coral–dinoflagellate symbioses are strongly temperature dependent. Global warming and the resultant coral bleaching are major threats to corals globally, but we speculate that ongoing OA is another potential stressor (Fig. 4b). One modelling study predicted that in the worst-case scenario, living coral communities will disappear from the coastal regions of Japan before the mid-21st century in response to the simultaneous degradation of their living conditions at both higher and lower latitudes, with acidification in the north and warming in the south^32^. Many coral reefs, including those at Chichijima and Kikaijima, receive good circulation from open ocean seawater. Open oceans will acidify more rapidly than inner-reef environments, which are characterized by the long residence time of the seawater, and are buffered by the dissolution of reef sediments, mitigating OA^5^. If the sensitivity of the Chichijima and Kikaijima corals to OA can be regarded as representative of the sensitivity of other corals, many coral reefs surrounding oceanic islands in the subtropics may be in more danger than originally thought. However, we note that there are large uncertainties in predicting the thresholds for each coral colony and each coral reef community on both the global and regional scales. This is because global and local stressors (e.g., OA, global warming, destructive fishing, sediment influx) interact in a highly complex ways, and the coral response can vary within and among coral species^4, 31^. For example, massive *Porites* coral in Guam^33^ display high sensitivity to the ^13^C Suess effect (with a large reduction in δ^13^C_coral_), but little sensitivity to OA (with a slight reduction in δ^11^B). This suggests that corals living in natural environments respond differently to OA, depending on the environmental factors affecting individual reefs. Therefore, more field-based studies with the boron technique are required to understand how corals have adapted to or are threatened by OA, before corals disappear under the impact of physical, chemical, and biological erosion^4, 31^.

## Methods

### Seawater CO_2_ chemistry estimation around Chichijima and Kikaijima

To describe the seawater CO_2_ chemistry, two of four measurable CO_2_ parameters must be determined. In this work, we used the fugacity of CO_2_ (*f*CO_2_) data stored in the Surface Ocean CO_2_ Atlas (SOCAT v.2)^34^. Total alkalinity (TA) was calculated from sea-surface salinity (SSS) using the relationship TA = (SSS/35) × 2295 μmol kg^−1^ (ref. 16). We used the *f*CO_2_ data obtained in a specific area (26.5–27.5°N, 125–145°E) and averaged it for each month for 1983–2011 (ref. 16). These discrete data were used to calculate the monthly DIC, Ω_arSW_, and pH_SW_. The values calculated for normalized DIC at a salinity of 35 (nDIC) were fitted to an empirical function for the time of the measurement, SST, and SSS with multi-parameter regression (Supplementary Section 2).

We also simulated a time series of seawater CO_2_ chemistry parameters for the 27°N,137°E grid point by combining the empirical function of nDIC obtained, TA, the monthly (1° × 1°)-resolution SST, and the SSS records from the Multivariate Ocean Variational Estimation (MOVE) system developed by the Meteorological Research Institute^35^. The validity of this estimate was confirmed by a comparison of the pH_SW_ and *p*CO_2SW_ time series obtained with the time series for 0.5° latitude, with a 2.5° longitude grid centred on Chichijima and Kikaijima (Supplementary Figs S4 and S5, Supplementary Section 2).

We extended the annual pH_SW_ estimate to before the industrial era using atmospheric *p*CO_2_ records from continuous observations at the MLO^17^ and the air trapped in the Antarctic ice sheet^18^. We determined the *b* value in the equation^2, 19, 30^:

1 $${{\rm{pH}}}_{{\rm{SW}}}={{\rm{pH}}}_{\mathrm{pre}-\mathrm{industrial}}-b\ast \,\mathrm{log}(\frac{p{{\rm{CO}}}_{2}({\rm{atm}})}{280})$$

we used two boundary conditions, pH_SW_ and atmospheric *p*CO_2_, for 1994 (8.125 and 358.8 μatm, respectively) and the preindustrial era (8.202 and 277.6 μatm, respectively)^3, 17, 18^ to determine the constant *b* (Fig. 2d). We used an increase in anthropogenic DIC of 50 μmol kg^−1^ for the subtropical North Pacific since the preindustrial era^3^ for the calculation, and determined *b* to be 0.7. We calculated this while keeping SST and SSS constant for all the annual pH_SW_ and *p*CO_2SW_ calculations before the industrial era, because we confirmed that their effects on the pH_SW_ estimated for the years 1911–1994 were negligibly small (Supplementary Section 3 and Supplementary Fig. S6).

### Geographic and ecological features of the study areas

Massive *Porites* coral was found at a water depth of 5.6 m in Miyanohama inlet, located on the north coast of Chichijima Island (Supplementary Fig. S7)^10^. The coral reef of Ogasawara Archipelago, including Chichijima, is categorized as an apron reef, i.e., the coastal area is limited and lacks a reef-flat system. Miyanohama inlet is located in the west of Anijima Strait and receives good water circulation from the open ocean. There is no river flowing into Miyanohama. The inclination is gentle inside the inlet, but becomes steep at its mouth. The corals in this region grow on volcanic basement rock or dead corals, with a moderate coral cover (~50%) and high diversity, including massive Merulinidae corals (e.g., *Platygyra daedalea*, *Leptoria phrygia*, *Goniastrea pectinata*), branching/encrusting Acroporidae corals (e.g., *Acropora florida*, *Acropora hyacinthus*, *Acropora gemmifera*), massive Poritidae corals (e.g., *Porites lutea*), and massive/encrusting Oculinidae corals (e.g., *Galaxea fascicularis*)^36^.

Another massive *Porites* coral was found at a water depth of 3.5 m, offshore from Arakizaki point, located on the south west coast of Kikaijima Island (Supplementary Fig. S7)^11^. The coral reef of Kikaijima is a small reef-flat system, almost all of which dries up on the ebb tide^37, 38^. As a result, the coral colony has developed in the limited area of reef slopes surrounding the island, with moderate coral cover (5–50%)^39^. There is no river on the surface of Kikaijima Island because the bedrock is composed of highly permeable calcium carbonate. The living coral reef assemblage offshore from Arakizaki has not been described, but according to observations at water depths of 1–5 m on the northeast side of the island (Shidooke)^38^, it is highly diverse, including branching Pocilloporidae corals (e.g., *Pocillopora verrucosa*), branching/tabulate/encrusting Acroporidae corals (e.g., *Acropora palifera*, *Acropora gemmifera*, *Acropora monticulosa*, *Acropora digitifera*), massive Poritidae corals (e.g., *Porites lobata*), and massive Merulinidae corals (e.g., *Favites abdita*, *Favites pentagona*, *Goniastrea retiformis*). These assemblages are also seen on the exposed terrace of Holocene reefs near Arakizaki^37^. We monitored the variability of the water temperature at this location by attaching a temperature logger to the appropriate massive *Porites* sp. from summer 2009 to summer 2011. This showed good agreement with the variation in the open ocean SST (Supplementary Fig. S8), confirming that the study site receives good circulation of open ocean seawater.

### δ^11^B and δ^13^C analyses

For the geochemical analysis of coral skeletons, we used a massive *Porites* sp. collected at Chichijima in October 2002 (ref. 10) and another sample collected at Kikaijima in June 2009 (ref. 11) (Fig. 1). The methodology used to prepare the 3-year-resolution subsamples of the Chichijima coral is described by Kubota *et al*.^19^. Briefly, we drilled a coral skeleton slab along the major growth direction and obtained single-year-resolution subsamples for the years 1873–2002. We discarded the subsamples for the most recent 4 years because the Sr/Ca and U/Ca values showed anomalously high values^10^. We also discarded subsamples from before 1910 because there was a climatic regime shift in 1905–1910 (ref. 10). We mixed equal amounts of powdered subsample for each single year and prepared 3-year-resolution subsamples to measure δ^11^B for 1910–1998. To prepare the 3-year-resolution subsamples of the Kikaijima coral to make the δ^11^B measurements for 1910–2009, we drilled the coral skeleton along the major growth direction and homogenized it. Typically, we used 3–6 mg of carbonate for the δ^11^B measurements. After removing the organic matter with 30% H_2_O_2_, we purified the boron using cation- and anion-exchange resin columns^40^ and dissolved the samples with a mixed acid composed of 0.15 M HNO_3_, 0.05 M HF, and 0.1% mannitol to obtain a solution of 75 ppb boron. We measured δ^11^B in both the Chichijima coral and Kikaijima coral with a multi-collector inductively coupled plasma mass spectrometer (MC-ICPMS; Thermo Finnigan NEPTUNE) installed at the Kochi Core Center (KCC), Japan, against the isotopic reference NIST-SRM 951, with a standard-sample bracketing technique under wet plasma conditions. We used the method of Foster^41^ to optimize the operating conditions for MC-ICPMS. All the δ^11^B values reported here are the averages of duplicate analyses (Supplementary Table 1). We compared the newly obtained δ^11^B data for the Chichijima coral with those measured with thermal ionization mass spectrometry (TIMS; Thermo Finnigan Triton) at KCC that were reported previously by Kubota *et al*.^19^, confirming the good reproducibility of the two different methods (MC-ICPMS and TIMS) (Supplementary Fig. S9 and Supplementary Table 1). The δ^11^B value of the international carbonate standard JCp-1, a *Porites* coral skeleton collected at Ishigakijima Island, determined with MC-ICPMS, was 24.44 ± 0.24‰ (2σ, n = 91), which was consistent with the previously reported value of 24.28 ± 0.14‰ (2σ, n = 14) determined with TIMS (Supplementary Fig. S9)^19^.

To determine the δ^13^C of the coral skeleton, the sub-monthly δ^13^C data for the Chichijima coral for 1910–1998, reported by Felis *et al*.^1^, were used and new measurements were made for the Kikaijima coral with an isotope ratio mass spectrometer (Thermo Fisher Scientific; Delta V plus) installed at the Atmosphere and Ocean Research Institute, Japan. All the isotope values are reported with respect to Pee Dee Belemnite (PDB) based on an NBS-19 value of 1.9‰. All the reported δ^13^C values are the averages of duplicate analyses (Supplementary Table 1). The repeated analysis of an in-house standard yielded an external reproducibility for the δ^13^C measurements of better than 0.13‰ (1σ, N = 123).

### ΔpH calculation from pH_CF_

We used a previously reported δ^11^B-pH_CF_ equation^26, 28, 29^ to determine the relationship between coral calcification and OA.

2 $${{\rm{pH}}}_{{\rm{CF}}}={\rm{p}}{K}_{B}-\,\mathrm{log}(\frac{{{\rm{\delta }}}^{11}{{\rm{B}}}_{{\rm{SW}}}-{{\rm{\delta }}}^{11}{{\rm{B}}}_{{\rm{carbonate}}}}{{{\rm{\alpha }}}_{3\mbox{--}4}\ast {{\rm{\delta }}}^{11}{{\rm{B}}}_{{\rm{carbonate}}}-{{\rm{\delta }}}^{11}{{\rm{B}}}_{{\rm{SW}}}+{10}^{3}\ast ({{\rm{\alpha }}}_{3\mbox{--}4}-1)})$$

3 $${\rm{\Delta }}\mathrm{pH}={{\rm{pH}}}_{{\rm{CF}}}-{{\rm{pH}}}_{{\rm{SW}}}$$

Here, pH_CF_ and pH_SW_ are the pH of the calcification fluid and of seawater, respectively; δ^11^B_SW_ is the global average δ^11^B of seawater (39.61‰; ref. 42); and α3–4 is the fractionation factor (1.0272; ref. 25). The dissociation constant for boric acid, p*K*_B_, is 8.60 at 24.6 °C (24.5 °C) and the salinity at Chichijima (Kikaijima) is 34.8 (34.5). We confirmed that the past changes in SST and SSS had negligible effects on the estimation of pH_CF_ (Supplementary Section 3 and Supplementary Fig. S6).

## Electronic supplementary material

Supplementary Information
