# Supplementary material for: Rapid decline in pH of coral calcification fluid due to incorporation of anthropogenic CO2
Source: Sci Rep. 2017 Aug 9;7:7694. doi: 10.1038/s41598-017-07680-0 (PMC5550433; doi:10.1038/s41598-017-07680-0)
Supplement: Supplementary file 1 — Supplementary Information [file 41598_2017_7680_MOESM1_ESM.pdf]

Supplementary Information

Rapid decline in pH of coral calcification fluid due to incorporation of anthropogenic CO_2

Kaoru Kubota, Yusuke Yokoyama, Tsuyoshi Ishikawa, Atsushi Suzuki, Masao Ishii

Supplementary Text

Supplementary Figures S1–S9

Supplementary Tables S1–S2

Supplementary References

Supplementary Text

1. Calculation of Ω_{CF}. Though $\delta^{11}B$ of coral skeleton can be used to estimate pH_{CF}, in order to fully describe CO_2 system in the calcification fluid more calculation is required (e.g., ref. 29,46). Thus from the following calculation we confirmed Ω_{CF} is dependent on pH_{CF}. All calculation were made in temperature of 25°C and salinity of 35 for simplicity. We used constants bellow:

$[B]_{SW}$ = 416 μmol/kg (ref. 47);

$[Ca]_{SW}$ = 10.2821 mmol/kg (ref. 48);

Solubility constant, K_{sp} for aragonite = $6.5 * 10^{-7}$ (ref. 49);

Dissociation constants of carbonic acid in the seawater: K_1 and K_2 from Lueker *et al.*[50];

Dissociation constants of boric acid in the seawater: K_B from Dickson[51].

We assume that calcium and boron concentration of the calcification fluid is same as seawater as its difference is less than 10% (ref. 6). Although how boron is incorporated into the aragonite skeleton from the seawater is still contested[46,52], we employed the relationship reported by Hemming & Hanson[53] which is based on empirical calibration of biogenically precipitated aragonite such as skeletons of corals and coralline algae living in the ocean. To employ Hemming & Hanson[53]'s model is reasonable, because there is a statistically significant correlation between B/Ca and $[B(OH)_4^-]/[HCO_3^-]$ in a boron concentration in the present seawater[46]. In this model, charged borate ion is selectively incorporated into aragonite lattice. Distribution constant, K_D is defined as:

$$K_D = \frac{[HBO_3^{2-}/CO_3^{2-}]_{solid}}{[B(OH)_4^-/HCO_3^-]_{liquid}} \cong \frac{[B/Ca]_{CaCO_3}}{[B(OH)_4^-/HCO_3^-]_{seawater}} \tag{S1}$$

where B/Ca is equivalent to $[HBO_3^-]/[CO_3^{2-}]$, because Ca and C are equimolar in $CaCO_3$. We made calculation using K_D value of $12*10^{-3}$ reported by Hemming & Hanson[53], which lies within ones recently determined by Holcomb *et al.*[46]: 1.4–52 $*10^{-3}$ and Mavromatis *et al.*[52]: 0.3–40 $*10^{-3}$.

For the Chichijima coral samples (3-year resolution), we newly measured B/Ca ratio by using a quadropole inductively coupled plasma mass spectrometry (Elan DRC, Perkin Elmer, USA). Average B/Ca value of the Chichijima coral is 0.536 mmol/mol during 1914–1997 (Table S2). For the Kikaijima coral, we used high-resolution B/Ca

records determined by using a laser ablation inductively coupled plasma mass spectrometry (Element XR, Thermo Scientific, Germany)[11]. From smoothed monthly-resolution data, we calculated 3-year averages for each data points that we measured $\delta^{11}B$. Average B/Ca value of the Kikaijima coral is 0.457 mmol/mol during 1911–2006 (Table S2).

Saturation state of the calcification fluid with respect to aragonite, $\Omega_{CF} = [Ca]_{CF}[CO_3^{2-}]_{CF}/K_{sp}$, is calculated to be 20.1–31.7 (Table S2), which is consistent with the previous estimation for *Favia* coral[6]. Through these calculations, we confirmed that there is a clear positive relationship between pH_{CF} and Ω_{CF} (Fig. S2). Therefore we concluded that pH_{CF} decrease would lead to Ω_{CF} decrease.

2. Further verification of pH_{SW} estimation using SOCAT fCO_2 data for the vicinity of Chichijima. To see how much pH_{SW} and $pCO_{2\,SW}$ time series for the grid point at 27°N, 137°E (Method) represent those of the ocean in the vicinity of Chichijima (27.1°N, 142.2°E) and Kikaijima (28.3°N, 130.0°E), as there are zonal distance of about 500–700 km between grid points, we estimated pH_{SW} and $pCO_{2\,SW}$ using SOCAT fCO_2 version 2 data closer to the islands[34]. We extracted the data from 1.0° latitude by 5.0° longitude grids centered on Chichijima and Kikaijima (Fig. S4). We calculated TA from the equation: SSS/35×2295 μmol kg^{-1} (ref. 16). In the calculation, we used ancillary *in situ* data of SST and SSS to each fCO_2 data-point. For grid points where SSS data was not available, we imported climatological salinity values from the World Ocean Atlas 2005. We calculated DIC using CO2Calc software, version 1.0 (ref. 54). We normalized DIC at salinity of 35 (nDIC) and fitted it to an empirical function of the timing of measurement (year), SST, and SSS by multi-parameter regression[14]:

$$\begin{aligned} &nDIC = DIC * 35 / SSS \\ &= C_0 + C_1 * (year - 2000) + C_2 * (SST - 24.7) + C_3 * (SST - 24.7)^2 \\ &+ C_4 * (SST - 24.7)^3 + C_5 * (SSS - 34.5) + \varepsilon \end{aligned} \qquad \text{(S2)}$$

The terms $C_0 \sim C_5$ are coefficients of multiple regressions, and ε represents the residual of the fitting. The polynomial of (SST–24.7) in the equation exhibits strong correlation with nDIC and SST. From this calculation we obtained an empirical regression equation for Chichijima ($R^2 = 0.74$, n = 1,034) and Kikaijima ($R^2 = 0.93$, n = 4,401) using the

below parameters.

Parameter	Chichijima	Kikaijima
C_0	1958.9	1953.1
C_1	1.14	1.16
C_2	-2.05	-3.65
C_3	0.26	0.38
C_4	-0.05	-0.02
C_5	-12.0	-5.62

Root mean squares of ε are 6.7 and 2.5 μmol/Kg, respectively. We used SST and SSS of the Simple Ocean Data Assimilation (SODA v2.2.4; ref. 55) to estimate monthly values of nDIC for the years 1983–2008, and calculated pH_{SW} and $pCO_{2\,SW}$ from DIC and assuming TA using CO2Calc (Fig. S4). We made calculations of seawater CO_2 chemistry using the dissociation constants of carbonic acid given by Lueker *et al.*[50], that of hydrogen sulfate by Dickson[51], and the pH scale of the total hydrogen ion concentration. We compared obtained pH_{SW} and $pCO_{2\,SW}$ time series with those for the grid point at 27°N, 137°E (Method). We observed no significant difference between them (Fig. S5).

3. SST effects on pH calculation. As pH is temperature-dependent, we evaluated SST effect on pH_{SW} estimation. In all calculations, SSS was kept constant at the present climatological value (34.8), as it had variation less than 0.05 during 1911–1997 (ref. 10), which can be regarded negligibly small. We calculated SST in the past from monthly Sr/Ca records of the Chichijima coral using equations reported by Felis *et al.*[10] for the same coral colony, and used them as a representative of past SST variation in the western North Pacific[11]:

$$\mathrm{Sr/Ca} = \frac{10.33 - 0.051 * \mathrm{SST}}{10^3} \qquad \text{(S3)}$$

We calculated nDIC from obtained SST and SSS (34.8) using Eq. (S2), where the term

for the linear trend was removed to examine seasonal to inter-annual variations. Then, using CO2calc we calculated de-trended pH_{SW} in combination with assumed TA. We calculated averages for each 3-year period that corresponded to the same portions of the coral skeleton used for $\delta^{11}B$ analyses. The 3-year averaged SST anomaly had a standard deviation of 0.9 °C, and that of the pH_{SW} anomaly was only 0.006 (Fig. S6a,b). Therefore, owing to 3-year averaging, we confirmed that effects of SST changes in the past were negligible in pH_{SW} calculation.

In addition, we confirmed the negligible effect of past SST changes on pH_{SW} estimation through the calculation using independently estimated reanalysis SST record, the extended reconstructed global sea surface temperature (ERSST version 4; ref. 56). The 3-year averaged SST anomaly had a standard deviation of 0.2 °C, and that of the pH_{SW} anomaly was only 0.002 (Fig. S6c,d).

Similarly, SST and SSS effects on pH_{CF} calculation were negligibly small as a standard deviation of pK_B change is less than 0.01.

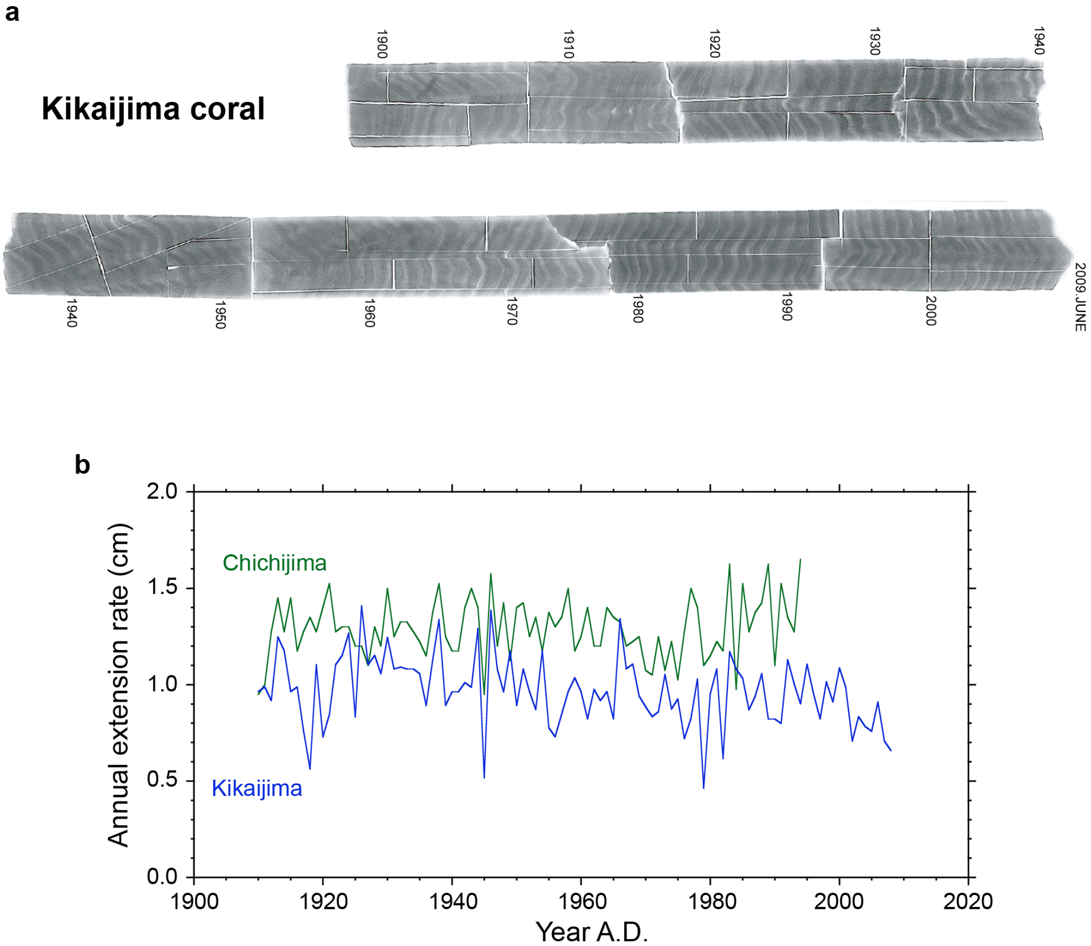

Figure S1. (**a**) A X-ray image of the Kikaijima coral. (**b**) Annual extension rates of the Chichijima and the Kikaijima coral along the major growth direction. Data of the Chichijima coral is previously published from Felis *et al.*[10] and that of the Kikaijima coral is calculated from distances of adjoining Sr/Ca peaks in winter measured by a laser ablation ICPMS[11].

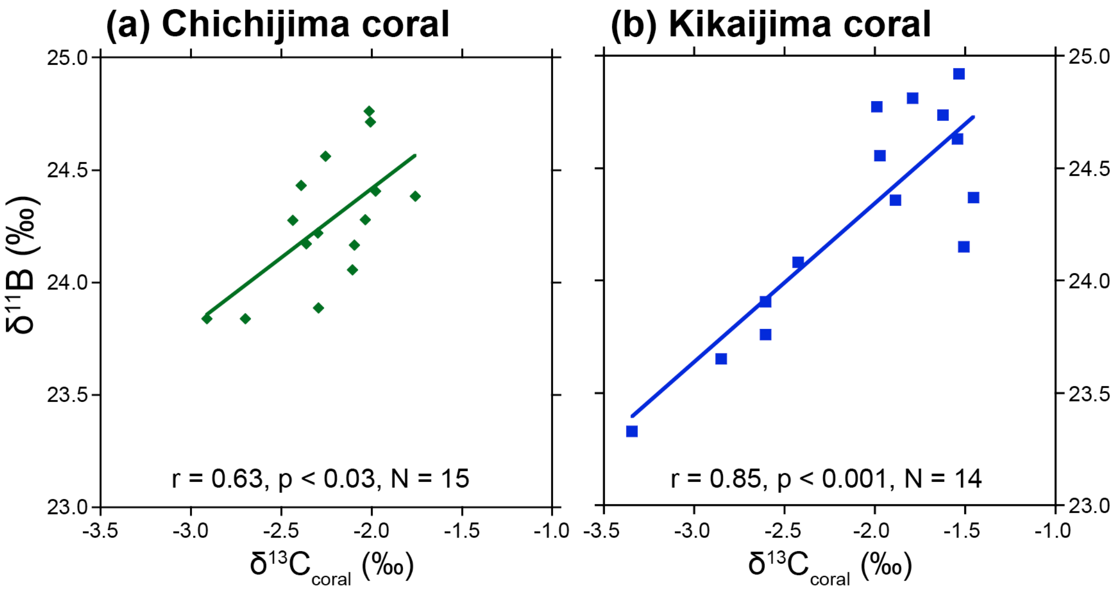

Figure S2. Linear regression of $\delta^{13}C$ versus $\delta^{11}B$ of (**a**) the Chichijima coral and (**b**) the Kikaijima coral. Correlation coefficient and probability values are shown.

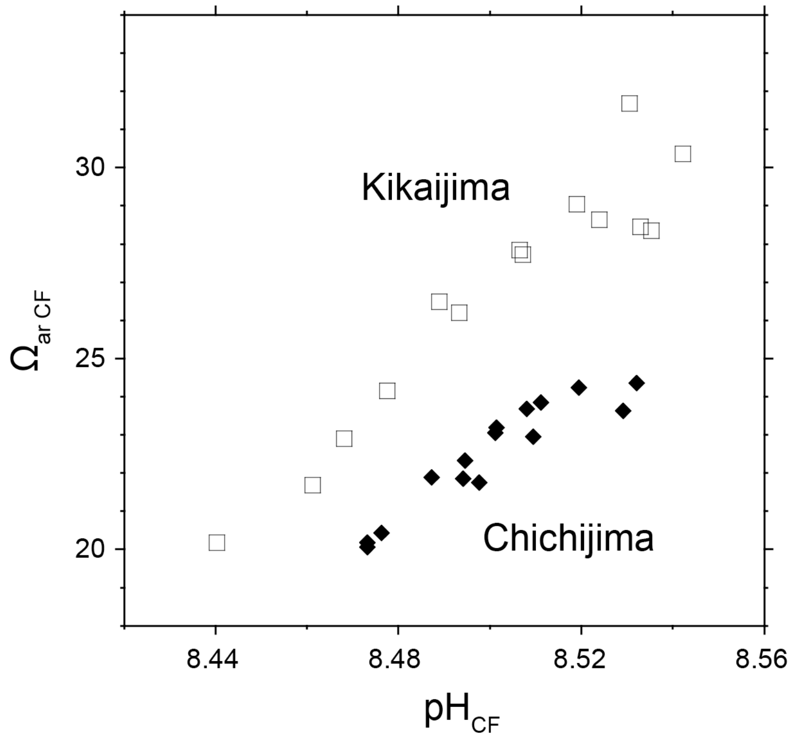

Figure S3. A cross-plot of pH and $\Omega_{aragonite}$ of the calcification fluid (the Chichijima coral, closed diamonds; the Kikaijima coral, open rectangles). CF, calcification fluid.

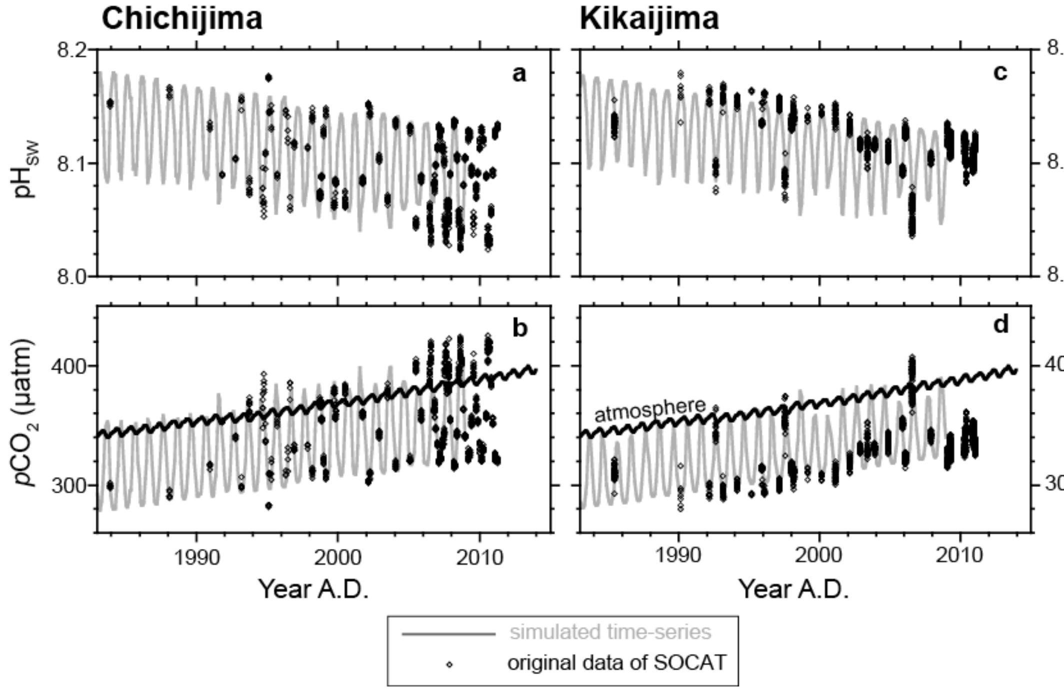

Figure S4. (**a**) pH_{SW} and (**b**) $pCO_{2\,SW}$ calculated from SOCAT fCO_2 (diamonds) and simulated time series (gray lines) using SODA SST and SSS for the years 1983–2008. Atmospheric pCO_2 is shown by the black line in **b** and **d**.

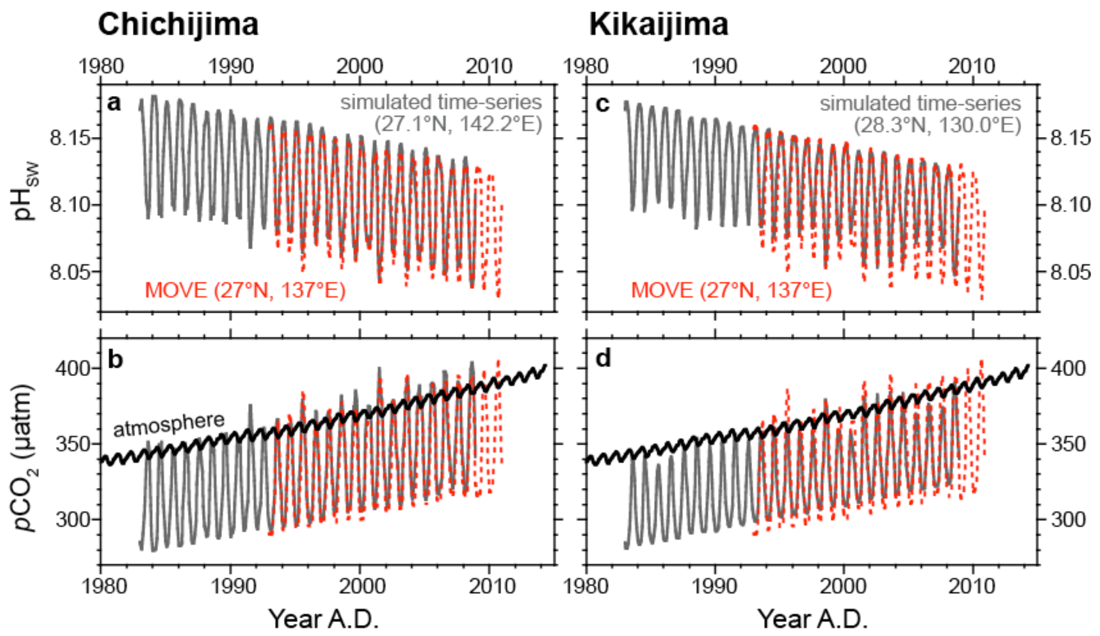

Figure S5. Comparison of (**a**) pH_{SW} and (**b**) $pCO_{2\,SW}$ time series simulated at 27°N, 137°E (for the years 1993–2010, red dashed lines) and those of the restricted areas around Chichijima and Kikaijima (for the years 1983–2008, gray lines). Atmospheric pCO_2 is shown by the black line in **b** and **d**. MOVE, multivariate ocean variational estimation system.

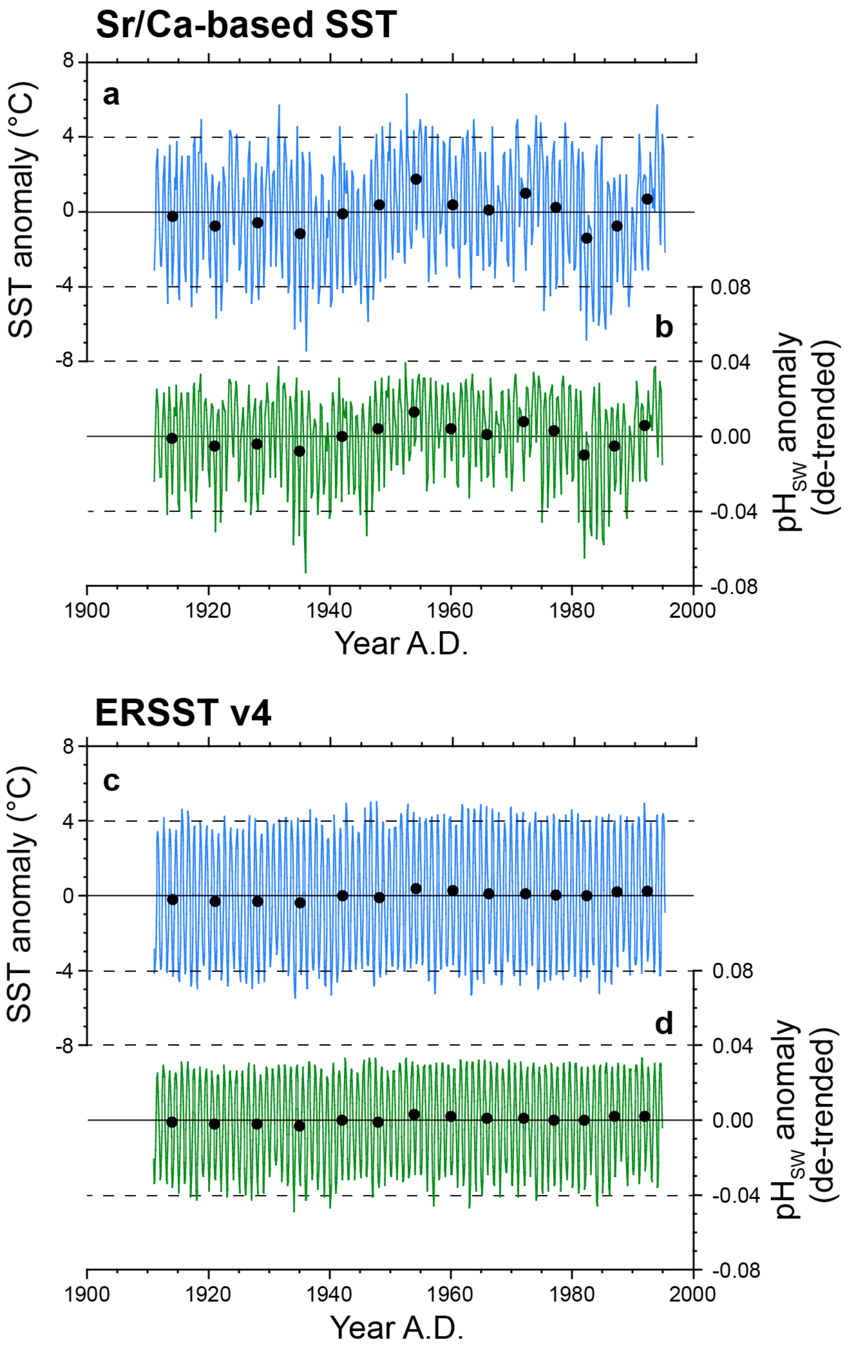

Figure S6. Monthly mean (**a**) SST (ºC) and (**b**) resulting pH_{SW} anomalies estimated from Sr/Ca paleo-thermometer of the Chichijima coral for the years 1911–1994 (lines). Black circles represent 3-year averages that correspond to portions of skeletons for which $\delta^{11}B$ were analyzed. (**c,d**) Same as (a,b) but estimated from reanalysis temperature record from ERSST version 4 at 28°N 142° E (ref. 56).

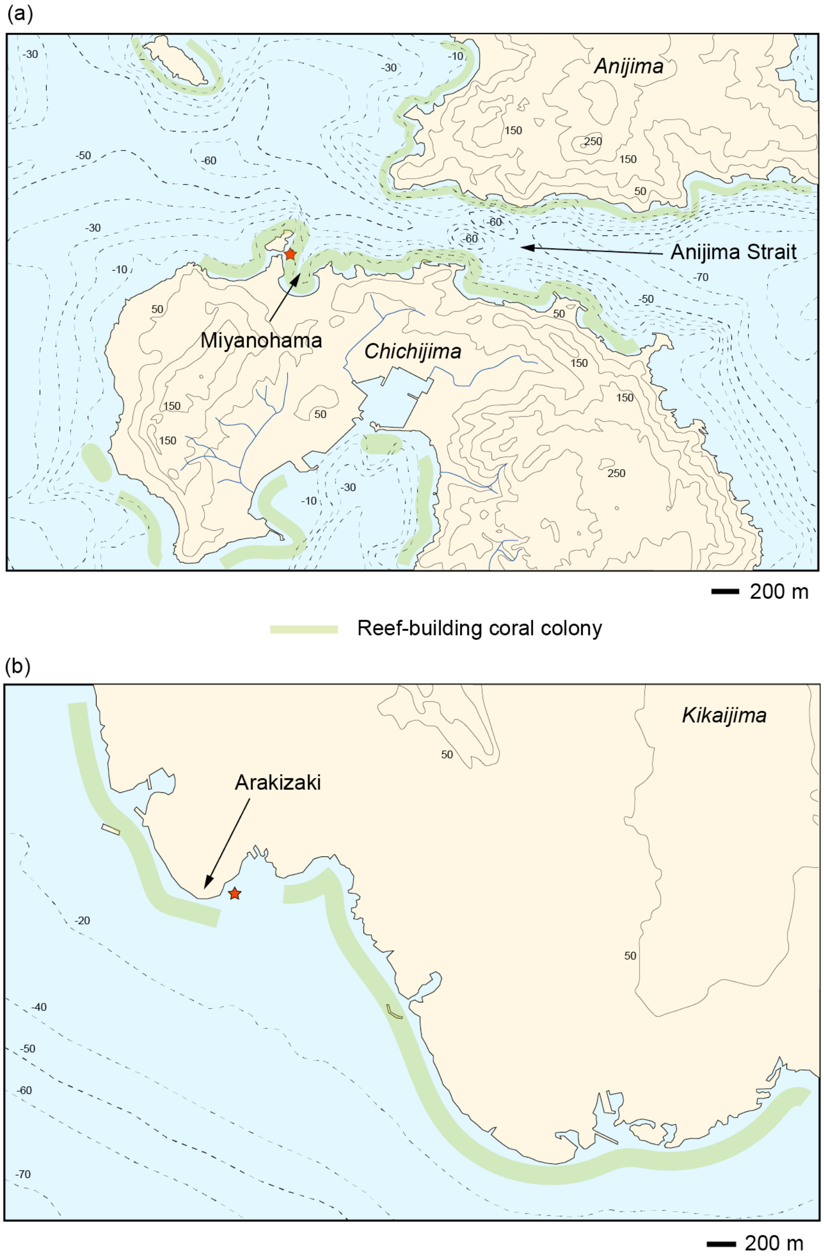

Figure S7. Geographical map of study areas in (**a**) Chichijima[57] and (**b**) Kikaijima[58]. Stars indicate positions of collected massive *Porites* spp.

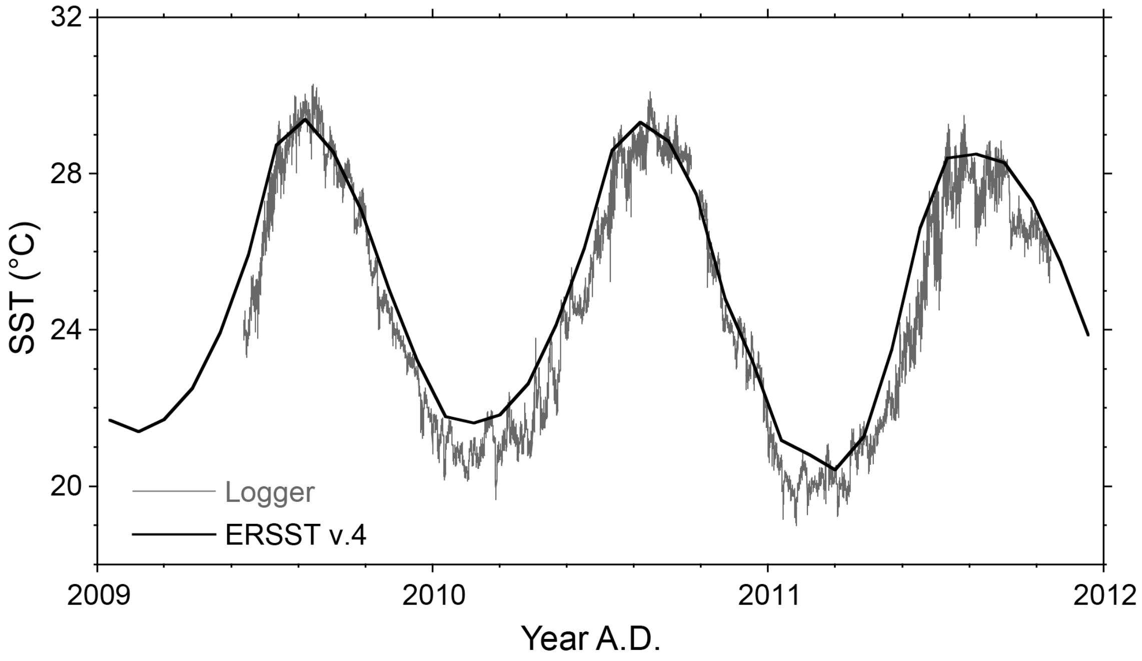

Figure S8. Comparison between *in situ* seawater temperature measured every hour using a data logger fixed to the Kikaijima coral (thin gray line) and gridded data sets of global SST based on marine observational records at 28°N 130°E (ERSST version 4, thick black line)[11,56]. A good similarity between the two indicates that the coral was living in the environment directly exposed to open-ocean.

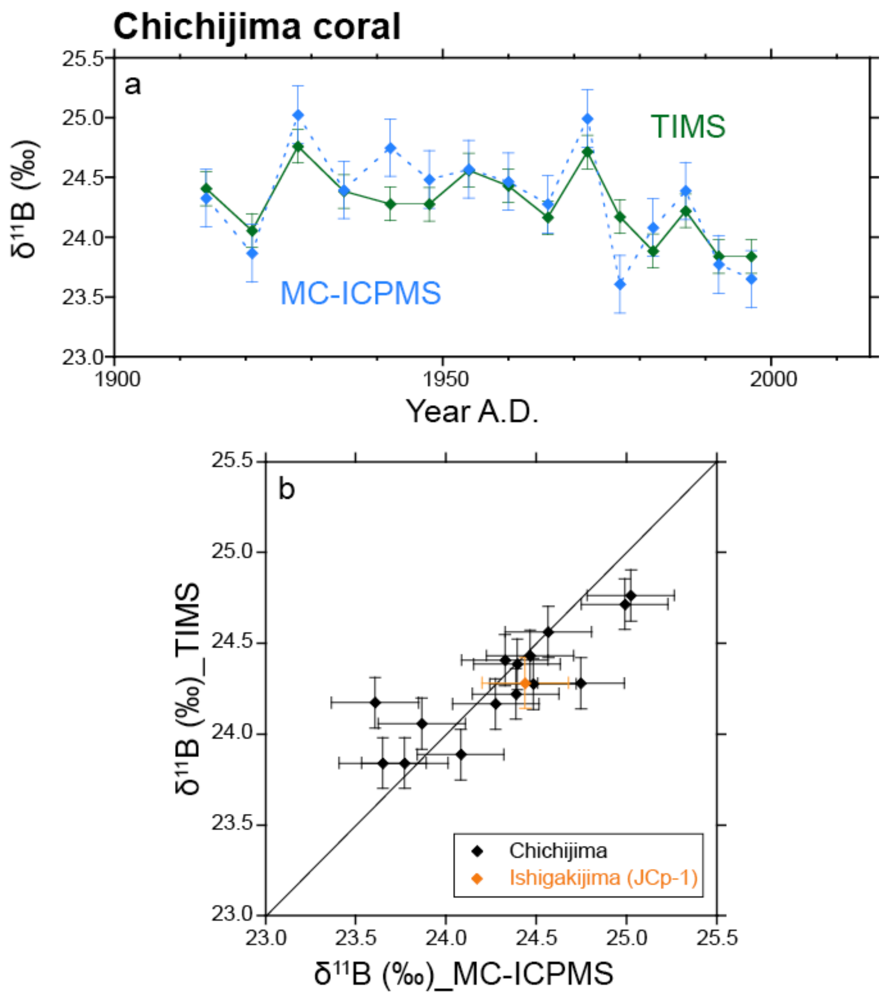

Figure S9. (**a**) Comparison of temporal $\delta^{11}B$ variation of the Chichijima coral samples since 1914 determined by MC-CIPMS and TIMS (see Table S1). (**b**) Relationship between $\delta^{11}B$ data obtained by MC-ICPMS and TIMS for the same set of *Porites* coral samples including JCp-1 and the ones shown in (a).

Supplementary Table 1. Measured $\delta^{11}B$ values for Kikaijima and Chichijima corals using MC-ICPMS and TIMS, calculated pH_{CF} from $\delta^{11}B$ values, estimated pH_{SW}, and measured $\delta^{13}C$ values.

Kikaijima		$\delta^{11}B$ (‰)					$\delta^{13}C$ (‰)		
**Years (AD)**	**Sample Name**	**#1**	**#2**	**Ave.**	**pH_{CF}**	**pH_{SW}**	**#1**	**#2**	**Ave.**
2005–2007	KAR09_C2_A1_1	23.20	23.46	23.33	8.43	8.12	-3.3	-3.4	-3.3
2000–2002	KAR09_C2_A1_2	23.32	23.98	23.65	8.45	8.13	-2.8	-2.9	-2.9
1993–1995	KAR09_C2_A2	23.71	24.11	23.91	8.47	8.14	-2.7	-2.5	-2.6
1987–1989	KAR09_C2_A3	24.06	23.46	23.76	8.46	8.14	-2.6	-2.6	-2.6
1980–1982	KAR09_C2_A4_1	24.69[a]	24.90	24.81	8.53	8.15	-1.8	-1.8	-1.8
		24.76[a]							
1974–1972	KAR09_C2_A4_2	25.06	24.48	24.77	8.53	8.16	-2.0	-2.0	-2.0
1968–1970	KAR09_C2_B2a	23.82[a]	24.29	24.08	8.48	8.17	-2.6	-2.3	-2.4
		23.93[a]							
1963–1965	KAR09_C2_B2b	24.63	24.64	24.63	8.52	8.17	-1.5	-1.6	-1.5
1954–1956	KAR09_C2_B3b	24.07[a]	24.25	24.15	8.49	8.18	-1.4	-1.6	-1.5
		24.03[a]							
1945–1947	KAR09_C2_B7b	24.63	24.48	24.56	8.51	8.18	-1.9	-2.0	-2.0
1937–1939	KAR09_C2_C1	24.94[a]	24.61	24.74	8.52	8.18	-1.5	-1.8	-1.6
		24.79[a]							
1928–1930	KAR09_C2_C3	24.63	24.09	24.36	8.50	8.19	-1.8	-2.0	-1.9
1919–1921	KAR09_C2_C4	24.66	25.18	24.92	8.53	8.19	-1.2	-1.8	-1.5
1910–1912	KAR09_C2_D1	24.34	24.39	24.37	8.50	8.19	-1.5	-1.4	-1.5

Chichijima		$\delta^{11}B$ (‰)				
**Years (AD)**	**Sample Name**	**MC-ICPMS**	**TIMS[b]**	**pH_{CF}**	**pH_{SW}**	**$\delta^{13}C$ (‰)[c]**
1996–1998	OGA02_3Y_1997	23.65	23.84	8.47	8.12	-2.7
1991–1993	OGA02_3Y_1992	23.77	23.84	8.47	8.12	-2.9
1986–1988	OGA02_3Y_1987	24.39	24.22	8.50	8.13	-2.3
1981–1983	OGA02_3Y_1982	24.08	23.89	8.48	8.14	-2.3
1976–1978	OGA02_3Y_1977	23.61	24.17	8.49	8.14	-2.4
1971–1973	OGA02_3Y_1972	24.99	24.71	8.53	8.15	-2.0
1965–1967	OGA02_3Y_1966	24.28	24.16	8.49	8.16	-2.1

1959–1961	OGA02_3Y_1960	24.47	24.43	8.51	8.16	-2.4
1953–1955	OGA02_3Y_1954	24.57	24.56	8.52	8.16	-2.3
1947–1949	OGA02_3Y_1948	24.48	24.28	8.50	8.16	-2.4
1941–1943	OGA02_3Y_1942	24.75	24.28	8.50	8.17	-2.0
1934–1936	OGA02_3Y_1935	24.40	24.38	8.51	8.17	-1.8
1927–1929	OGA02_3Y_1928	25.02	24.76	8.53	8.17	-2.0
1920–1922	OGA02_3Y_1921	23.87	24.06	8.49	8.17	-2.1
1913–1915	OGA02_3Y_1914	24.33	24.41	8.51	8.17	-2.0

(a) The same sample solution was measured by MC-ICPMS twice.

(b) TIMS data are from Kubota *et al.*[19]

(c) Calculated using sub-monthly $\delta^{13}C$ records reported by Felis *et al.*[10]

Supplementary Table 2. Calculated CO_2 system parameters in the calcification fluid.

Years (AD)	Sample Name	pH_{CF}	B/Ca (mmol/mol)	$[B(OH)_3]_{CF}$ (μmol/kg)	$[B(OH)_4^-]_{CF}$ (μmol/kg)	$[CO_3^{2-}]_{CF}$ (μmol/kg)	$[HCO_3^-]_{CF}$ (μmol/kg)	DIC_{CF} (μmol/kg)	TA_{CF} (μmol/kg)	Ω_{CF}
Kikaijma										
2005–2007	KAR09_C2_A1_1	8.44	0.480	245	171	1272	4269	5542	6985	20.2
2000–2002	KAR09_C2_A1_2	8.46	0.482	240	176	1368	4373	5740	7283	21.7
1993–1995	KAR09_C2_A2	8.48	0.459	237	179	1524	4692	6216	7920	24.2
1987–1989	KAR09_C2_A3	8.47	0.468	239	177	1444	4545	5989	7611	22.9
1980–1982	KAR09_C2_A4_1	8.54	0.481	223	193	1788	4819	6606	8587	28.3
1974–1972	KAR09_C2_A4_2	8.53	0.475	223	193	1795	4865	6660	8648	28.5
1968–1970	KAR09_C2_B2a	8.49	0.436	234	182	1671	5013	6685	8538	26.5
1963–1965	KAR09_C2_B2b	8.52	0.457	226	190	1807	5000	6807	8804	28.6
1954–1956	KAR09_C2_B3b	8.49	0.448	233	183	1652	4906	6559	8394	26.2
1945–1947	KAR09_C2_B7b	8.52	0.443	227	189	1832	5127	6959	8980	29.0
1937–1939	KAR09_C2_C1	8.53	0.423	224	192	1999	5447	7446	9637	31.7
1928–1930	KAR09_C2_C3	8.51	0.442	230	186	1756	5058	6813	8755	27.8
1919–1921	KAR09_C2_C4	8.54	0.460	221	195	1916	5082	6997	9108	30.4
1910–1912	KAR09_C2_D1	8.51	0.445	230	186	1748	5028	6777	8712	27.7
Chichijima										
1996–1998	OGA02_3Y_1997	8.47	0.544	238	178	1266	3938	5204	6648	20.1
1991–1993	OGA02_3Y_1992	8.47	0.541	238	178	1273	3959	5232	6684	20.2

1986–1988	OGA02_3Y_1987	8.50	0.548	232	184	1372	4032	5403	6959	21.7
1981–1983	OGA02_3Y_1982	8.48	0.540	237	179	1289	3980	5269	6737	20.4
1976–1978	OGA02_3Y_1977	8.49	0.528	233	183	1408	4169	5578	7169	22.3
1971–1973	OGA02_3Y_1972	8.53	0.564	224	192	1490	4075	5565	7247	23.6
1965–1967	OGA02_3Y_1966	8.49	0.539	233	183	1378	4085	5464	7025	21.8
1959–1961	OGA02_3Y_1960	8.51	0.524	229	187	1504	4288	5793	7484	23.8
1953–1955	OGA02_3Y_1954	8.52	0.531	227	189	1529	4276	5805	7523	24.2
1947–1949	OGA02_3Y_1948	8.50	0.523	231	185	1455	4242	5697	7337	23.1
1941–1943	OGA02_3Y_1942	8.50	0.521	231	185	1463	4264	5726	7374	23.2
1934–1936	OGA02_3Y_1935	8.51	0.522	229	187	1494	4288	5782	7463	23.7
1927–1929	OGA02_3Y_1928	8.53	0.553	224	192	1537	4173	5710	7439	24.4
1920–1922	OGA02_3Y_1921	8.49	0.524	234	182	1381	4159	5540	7102	21.9
1913–1915	OGA02_3Y_1914	8.51	0.542	229	187	1447	4141	5588	7222	22.9

Supplementary References

46. Holcomb, M., DeCarlo, T. M., Gaetani, G. A., & McCulloch, M. Factors affecting B/Ca ratios in synthetic aragonite. *Chem. Geol.* **437,** 67–76 (2016).
47. Millero, F. J. The thermodynamics of the carbonate system in sea- water. *Geochim. Cosmochim. Acta* **43,** 1651–16 (1979).
48. Millero, F. J., Feistel, R., Wright, D. G. & McDougall, T. J. The composition of standard seawater and the definition of the reference-composition salinity scale. *Deep-Sea Res. I* **55,** 50–72 (2008).
49. Mucci, A. & Morse, J. W. The incorporation of Mg^{2+} and Sr^{2+} into calcite overgrowths: Influences of growth rate and solution composition. *Geochim. Cosmochim. Acta* **47,** 217–233 (1983).
50. Lueker, T. J., Dickson, A. G. & Keeling, C. D. Ocean pCO_2 calculated from dissolved inorganic carbon, alkalinity, and equations for K_1 and K_2: validation based on laboratory measurements of CO_2 in gas and seawater at equilibrium. *Mar. Chem.* **70,** 105–119 (2000).
51. Dickson, A. G. Thermodynamics of the dissociation of boric acid in synthetic seawater from 273.15 to 318.15 K. *Deep-Sea Res.* **37,** 755–766 (1990).
52. Mavromatis, V., Montouillout, V., Noireaux, J., Gaillardet, J. & Schott, J. Characterization of boron incorporation and speciation in calcite and aragonite from co-precipitation experiments under controlled pH, temperature and precipitation rate. *Geochim. Cosmochim. Acta* **150,** 299–313 (2015).
53. Hemming, N. G. & Hanson, G. N. Boron isotopic composition and concentration in modern marine carbonates. *Geochim. Cosmochim. Acta* **56,** 537–543 (1992).
54. Robbins, L. L., Hansen, M. E., Kleypas, J. A. & Meylan, S. C. CO2calc—A user-friendly seawater carbon calculator for Windows, Max OS X, and iOS (iPhone). *U.S. Geological Survey Open-File Report* 2010–1280 (2010).
55. Carton, J. A. & Giese, B. S. A Reanalysis of Ocean Climate Using Simple Ocean Data Assimilation (SODA). *Mon. Wea. Rev.* **136,** 2999–3017 (2008).
56. Huang, B. *et al.* Extended reconstructed sea surface temperature version 4 (ERSST.v4). Part I: upgrades and intercomparisons. *J. Clim.* **28,** 911–930 (2015).
57. Maritime Safety Agency. Submarine structural chart, bathymetric chart, report of survey (Chichijima), basic map of the sea in coastal waters, Scale 1:50,000. <http://www.jha.or.jp/shop/>, (1998) Date of access: 27/04/2017

58. Maritime Safety Agency. Submarine structural chart, bathymetric chart, report of survey (Eastern part of Amami Gunto), basic map of the sea in coastal waters, Scale 1:200,000. <http://www.jha.or.jp/shop/>, (1982) Date of access: 27/04/2017
